# Correction: Long non-coding RNA SNHG17 may function as a competitive endogenous RNA in diffuse large B-cell lymphoma progression by sponging miR-34a-5p

**DOI:** 10.1371/journal.pone.0317025

**Published:** 2024-12-31

**Authors:** Shengjuan Lu, Lin Zeng, Guojun Mo, Danqing Lei, Yuanhong Li, Guodi Ou, Hailian Wu, Jie Sun, Chao Rong, Sha He, Dani Zhong, Qing Ke, Qingmei Zhang, Xiaohong Tan, Hong Cen, Xiaoxun Xie, Chengcheng Liao

There are errors in the author affiliations. The correct affiliations are as follows:

Shengjuan Lu^2^‡, Lin Zeng^1^‡, Guojun Mo^3,4^‡, Danqing Lei^4^, Yuanhong Li^3^, Guodi Ou^5^, Hailian Wu^4^, Jie Sun^1^, Chao Rong^1^, Sha He^1^, Dani Zhong^1^, Qing Ke^1^, Qingmei Zhang^6,7^, Xiaohong Tan^1^, Hong Cen^1^, Xiaoxun Xie^6,7^*, Chengcheng Liao^1^*

**1** Department of Hematology/Oncology, Guangxi Medical University Cancer Hospital, Nanning, China, **2** Department of Pharmacy, Foresea Life Insurance Guangxi Hospital, Nanning, China, **3** Department of Pharmacy, The Second Affiliated Hospital of Guangxi Medical University, Nanning, China, **4** Life Sciences Institute, Guangxi Medical University, Nanning, China, **5** Pharmaceutical College, Guangxi Medical University, Nanning, China, **6** Department of Histology and Embryology, School of Pre-clinical Medicine, Guangxi Medical University, Nanning, China, **7** Key Laboratory of Early Prevention and Treatment of Regional High Frequency Tumor (Guangxi Medical University), Ministry of Education, Nanning, China
