# Supplementary figures and images for: Long non-coding RNA SNHG17 may function as a competitive endogenous RNA in diffuse large B-cell lymphoma progression by sponging miR-34a-5p
Source: PLoS One. 2023 Nov 21;18(11):e0294729. doi: 10.1371/journal.pone.0294729 (PMC10662735; doi:10.1371/journal.pone.0294729)

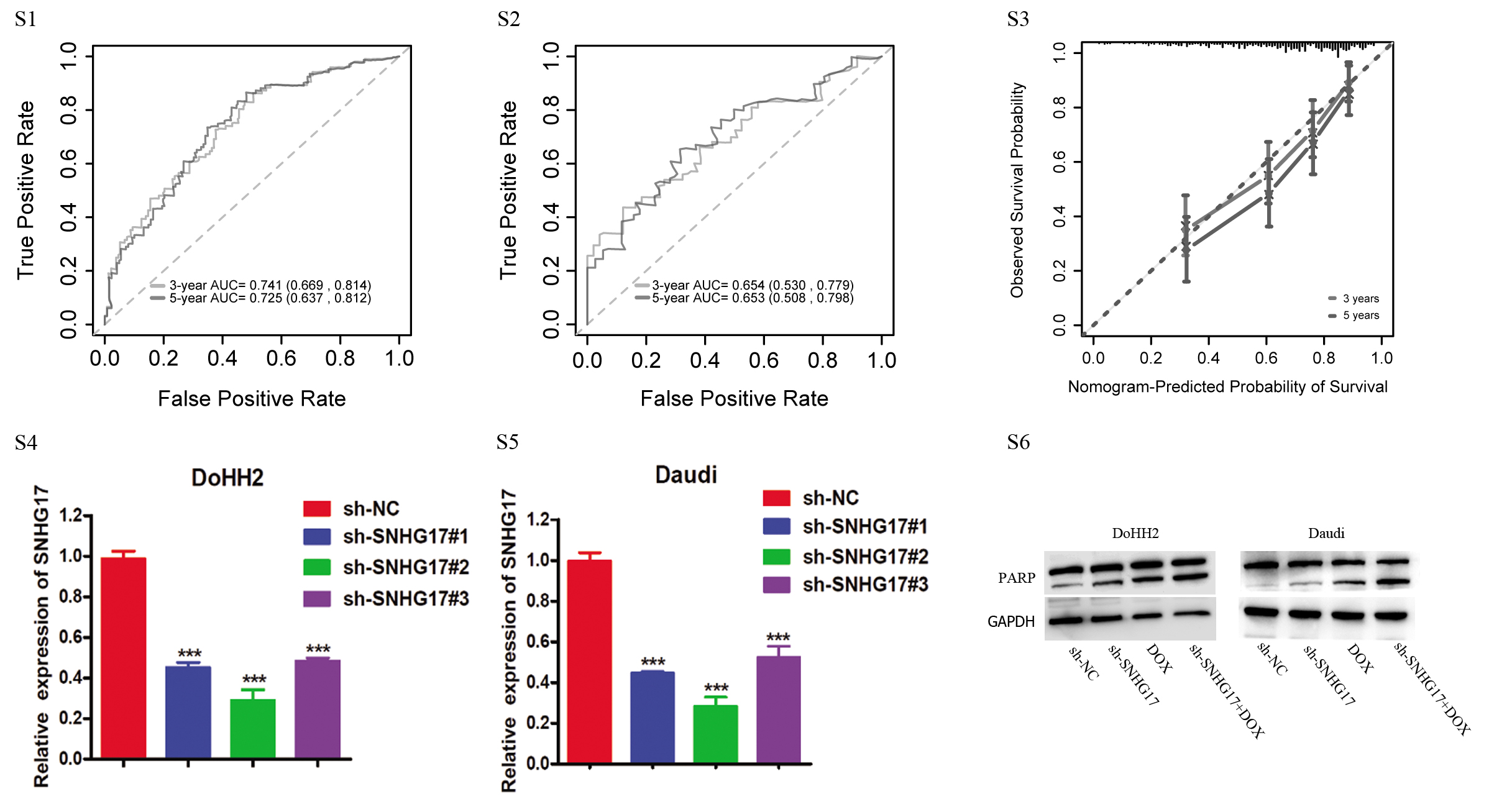

Supplement: S1 Data — (S1, S2) Time-dependent ROC curve analysis of Cox regression model in the test cohort. (S3) Nomograph calibration chart of 3- and 5-year OS probability.(S4) The knockdown efficiency of SNHG17 in DoHH2 cells was detected by qPCR. (S5) The knockdown efficiency of SNHG17 in Daudi cells was detected by qPCR.(S6) Apoptosis-associated protein PARP was examined using western blotting. *P < 0.05, **P < 0.01, ***P < 0.001. (JPG) [file pone.0294729.s001.jpg]
