# Supplementary material for: Long non-coding RNA SNHG17 may function as a competitive endogenous RNA in diffuse large B-cell lymphoma progression by sponging miR-34a-5p
Source: PLoS One. 2023 Nov 21;18(11):e0294729. doi: 10.1371/journal.pone.0294729 (PMC10662735; doi:10.1371/journal.pone.0294729)

Supplementary figure S6-Daudi-PARP

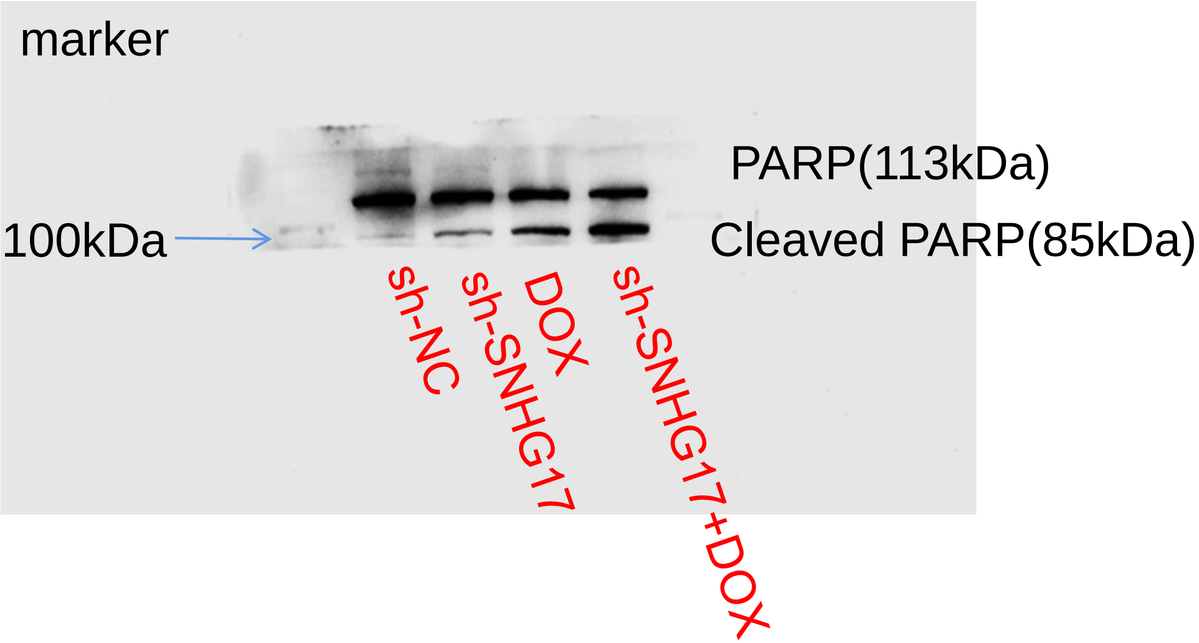

Supplementary figure S6-Daudi-GAPDH

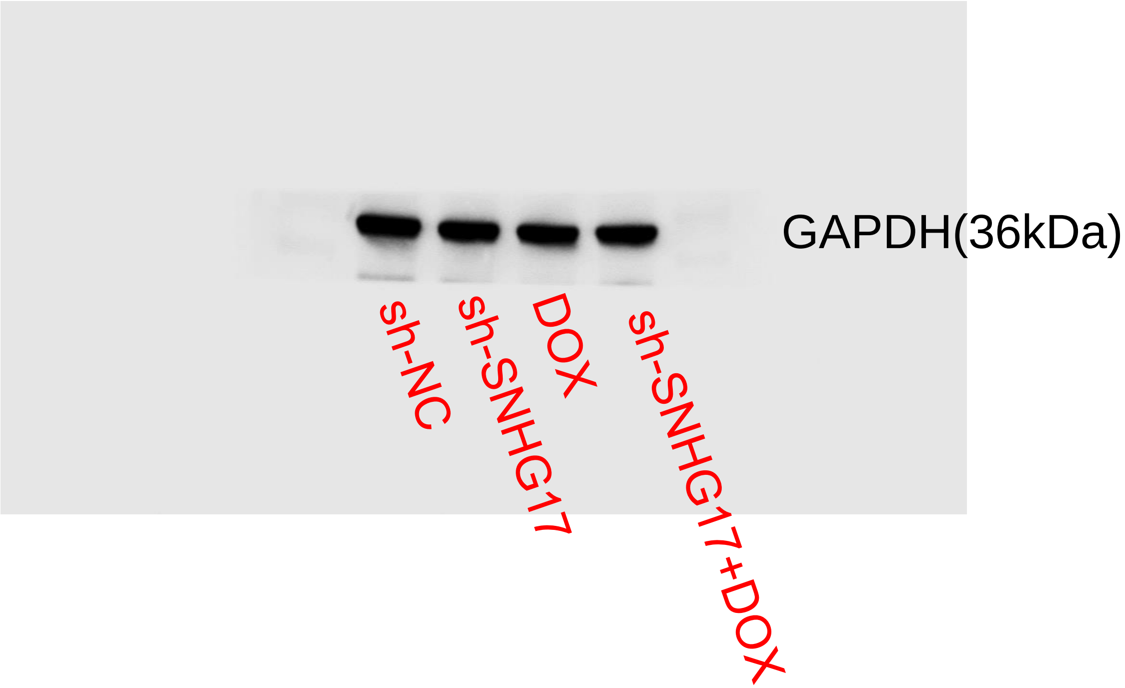

Supplementary figure S6-DoHH2-PARP

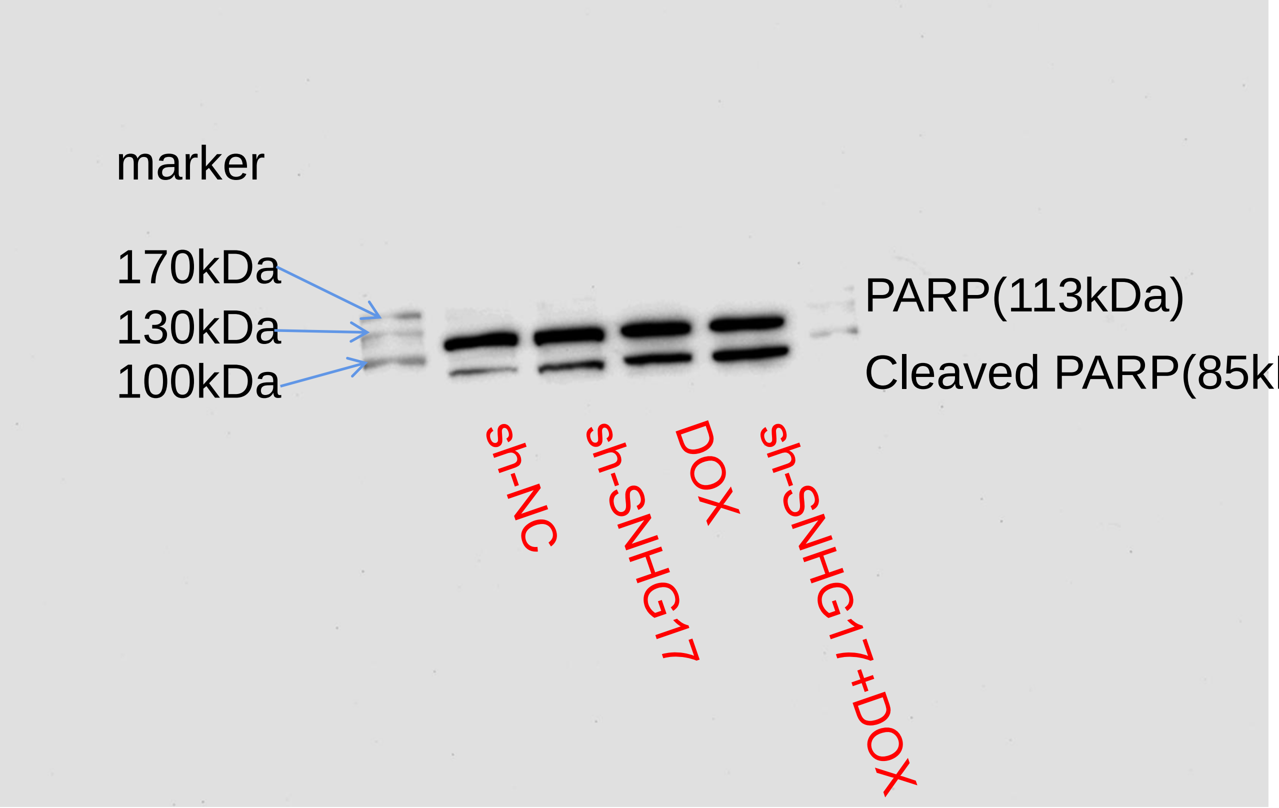

Supplementary figure S6-DoHH2-GAPDH

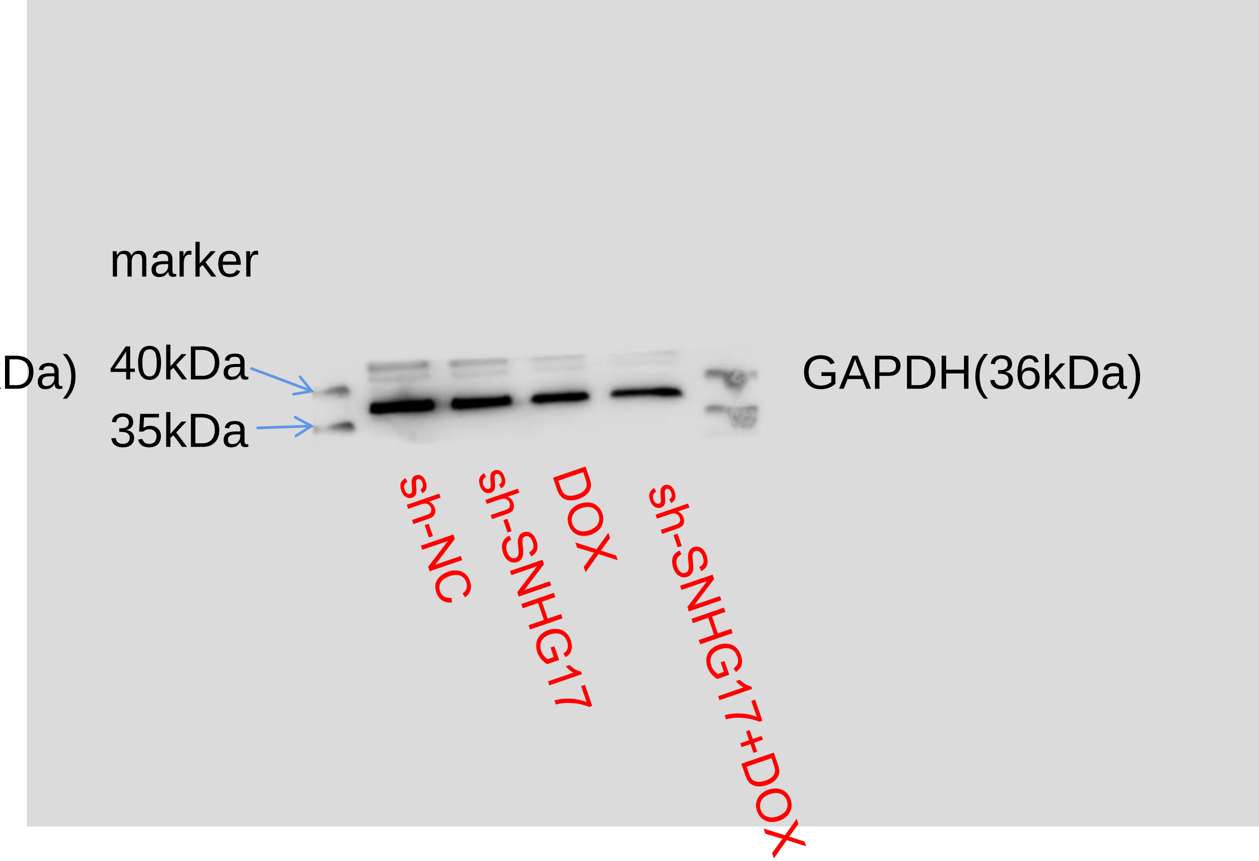

figure6-DⅠ-EZH2:

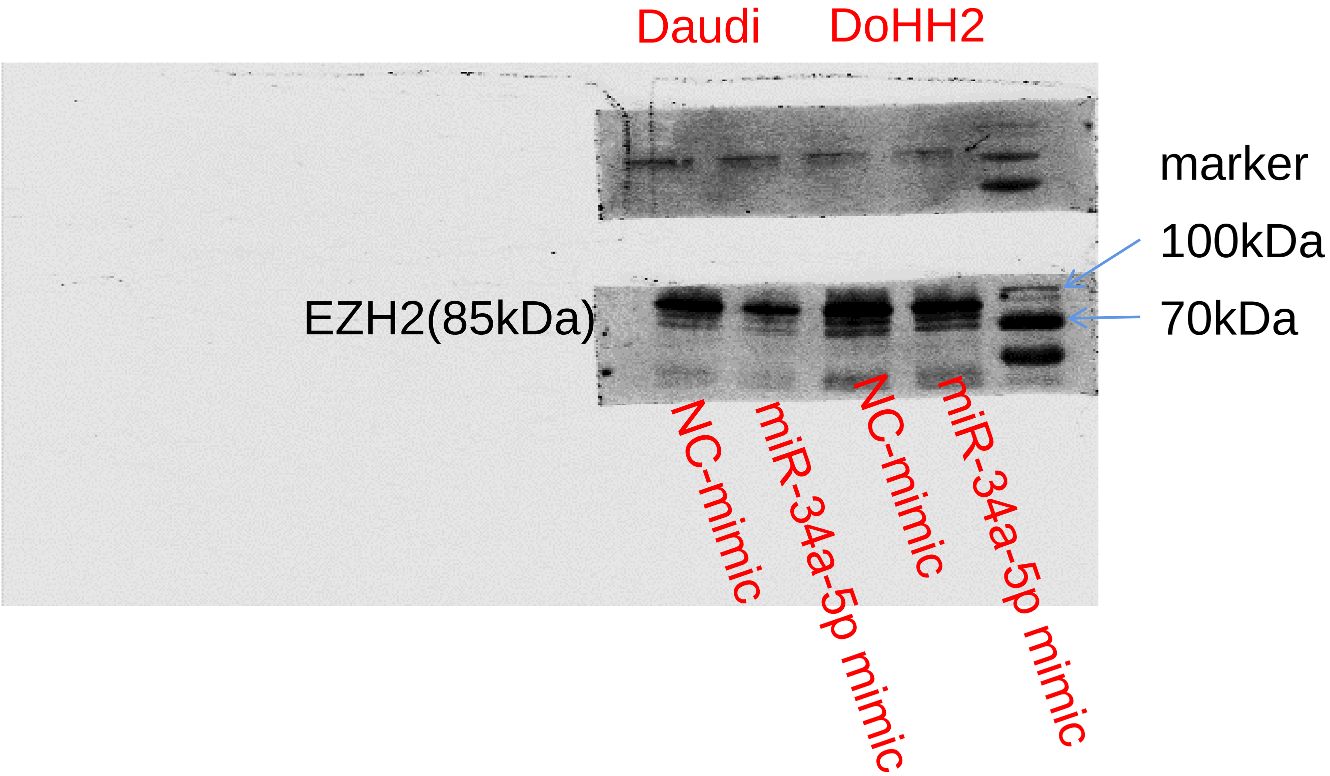

figure6-DⅠ-GAPDH:

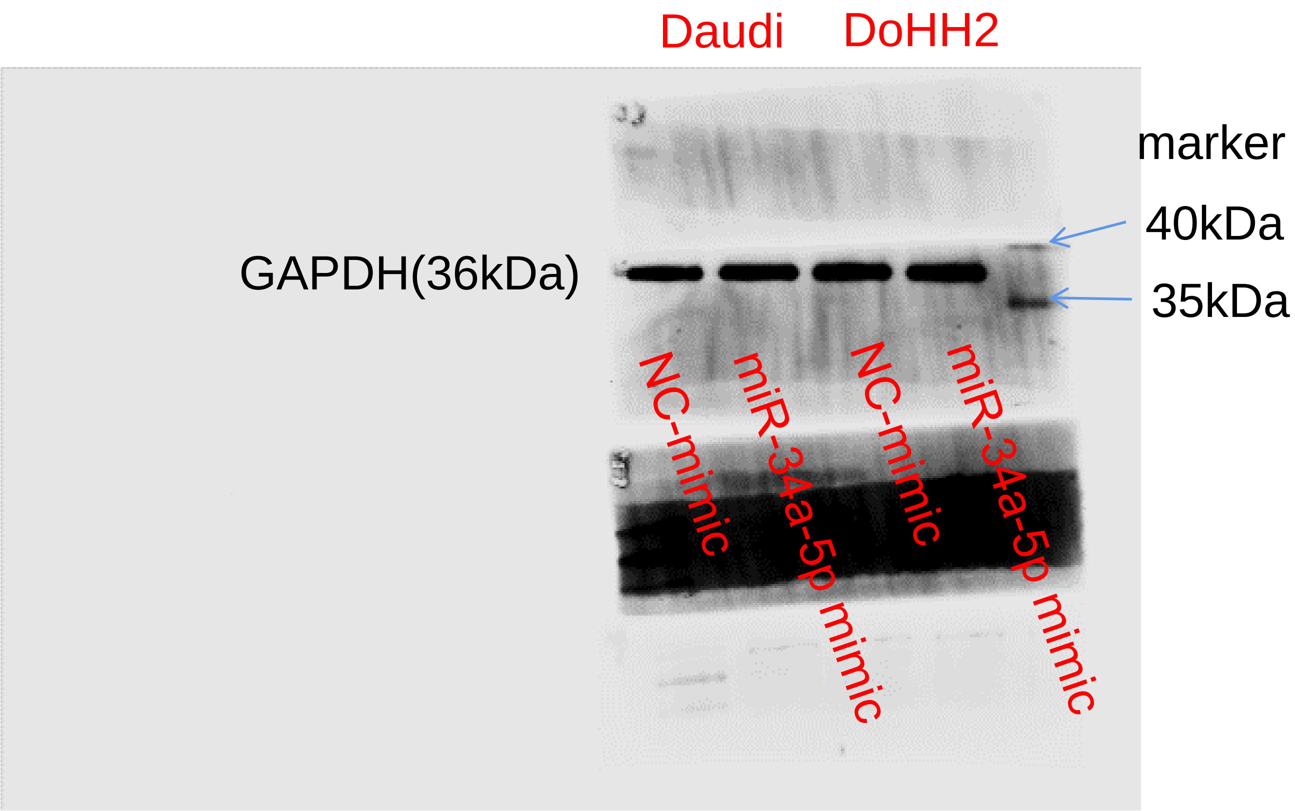

figure6-E-DoHH2-EZH2:

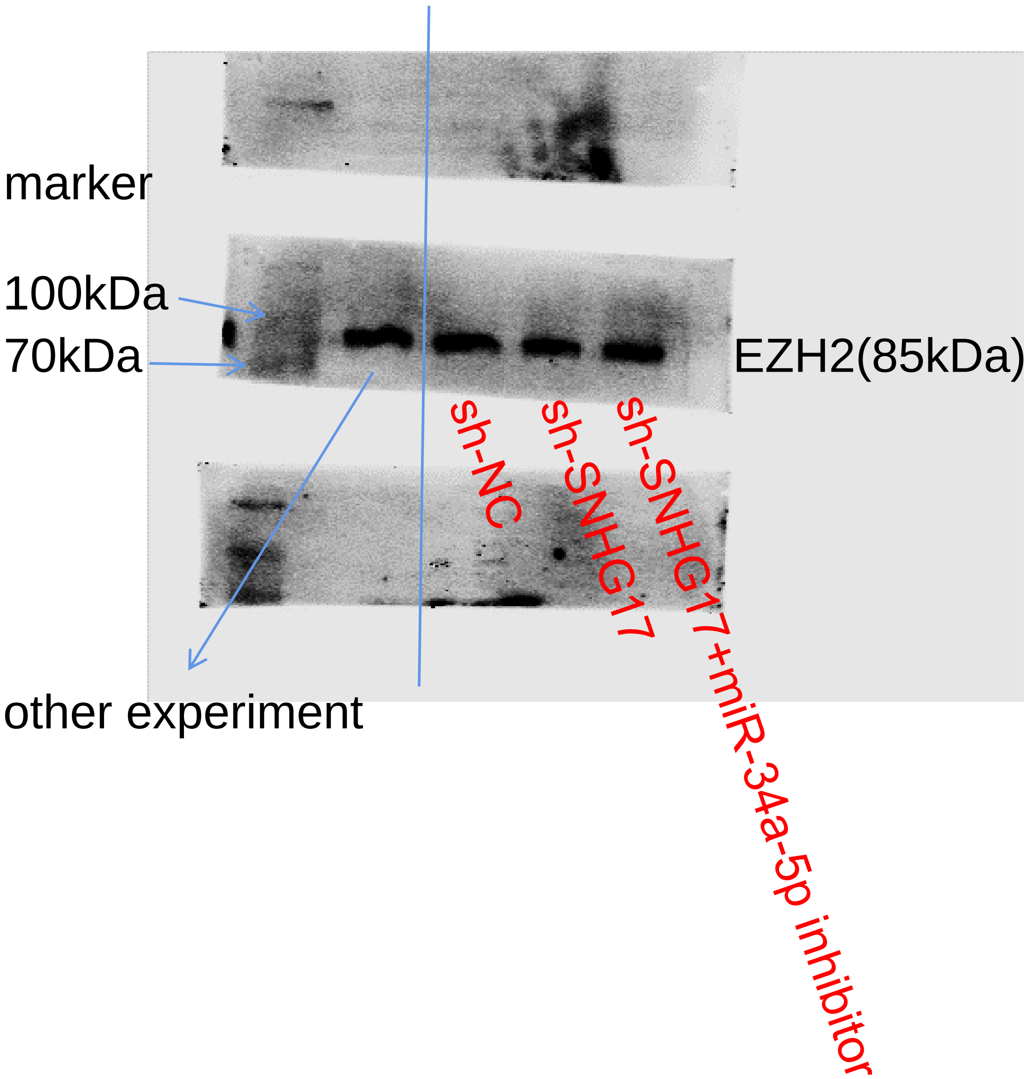

figure6-E-DoHH2-GAPDH:

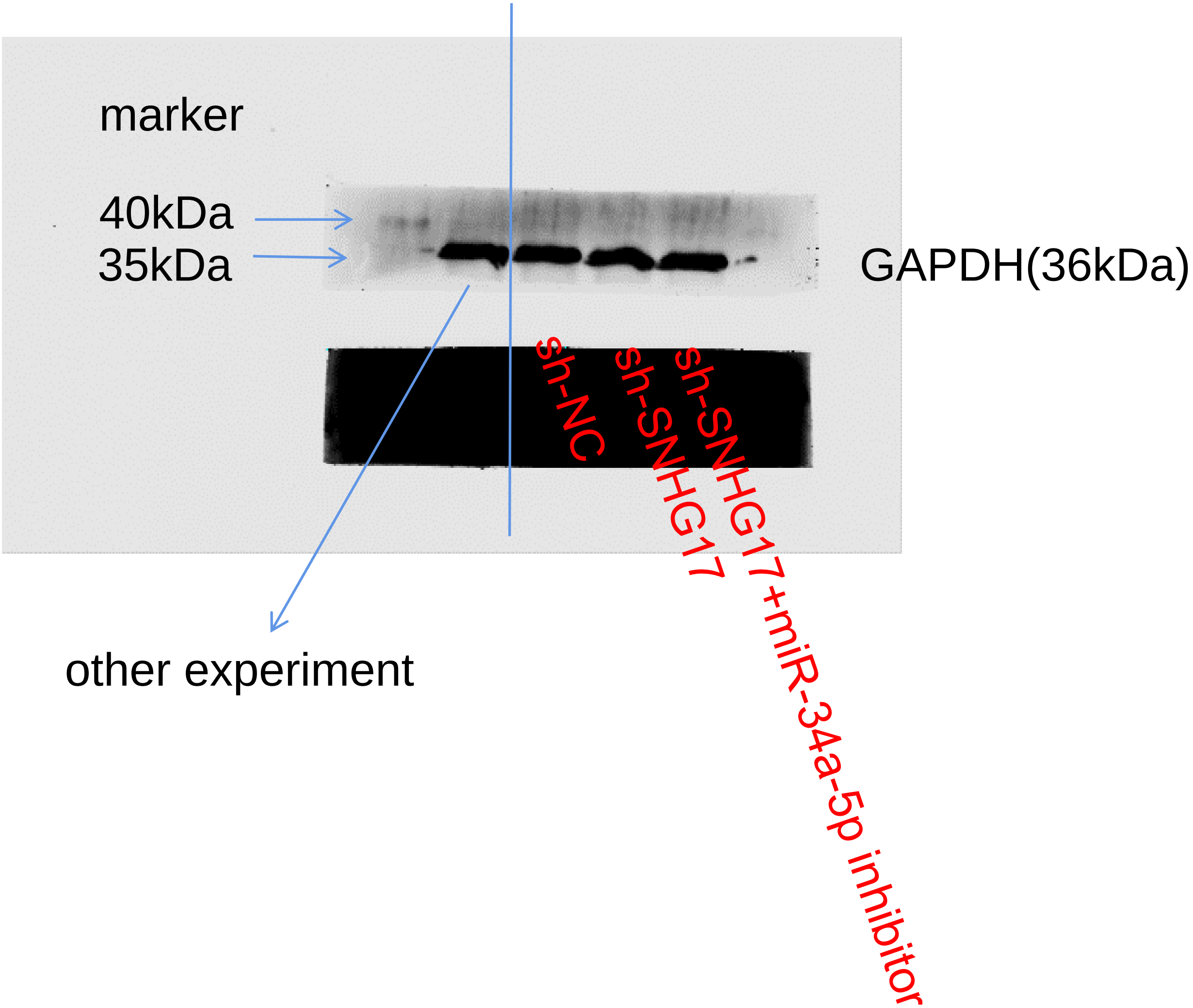

Supplement: S1 Raw images — (PDF) [file pone.0294729.s003.pdf]
