# Supplementary material for: Long non-coding RNA SNHG17 may function as a competitive endogenous RNA in diffuse large B-cell lymphoma progression by sponging miR-34a-5p
Source: PLoS One. 2023 Nov 21;18(11):e0294729. doi: 10.1371/journal.pone.0294729 (PMC10662735; doi:10.1371/journal.pone.0294729)
Supplement: Attachment — Submitted filename: reviewer requests.docx [file pone.0294729.s004.docx]

99 GSE10846 and GSE31312 datasets were downloaded from the Gene Expression Omnibus (GEO)

100 database. *describe at least the main features of the chosen datasets: i.e. disease, treatment, number of subjects*

123 serum. The cells were cultured in DMEM/ RPMI-1640 (Gibco, Waltham, MA, USA) supplemented

124 with 10% fetal bovine serum (Gibco) and 100 U/mL penicillin and streptomycin (Gibco) at 37 °C in

125 a 5% CO2 incubator. *Which kind of cells?*

168 detected using a chemiluminescence detection system (GE Healthcare, Chicago, IL, USA). The

169 primary antibodies used in this study were as follows: anti-EZH2 (1:2000,Abcam Technology Inc.),

170 anti-*PARP~~parp~~* (1:1500,Abcam Technology Inc.), and anti-caspase-3 (1:1500,Abcam Technology Inc.) and *Specify the name of ab clones.*

253 3.3 SNHG17 is highly expressed in DLBCL *patients’ tumor cells and* *in B-lymphoma* cell lines *(or DLBCL cell lines, as you call them in the latest part of the paper)*

541 Figure 2. SNHG17 is highly expressed in DLBCL *patients’ tumor cells and* *in B-lymphoma* cell lines. (A) Survival

542 analyses of *GSE10846 (n=*70*)* DLBCL patients with high and low expression of SNHG17 *~~in our cohort~~*.

545 patients with OS. (G) Expression of SHG17 in DLBCL patients*’* *tumor* and normal tissues*~~, tumor: diffuse~~*

*~~546 large B-cell lymphoma.~~  Explain better they are from CGA*

261 constructed DoHH2 and Daudi cells with SNHG17 knocked down by stably *~~transforming~~ transfecting* sh-

*-Fig 2 from I to J and its description are neither pertinent to point 3.3., nor relevant at all. At most you can enter them in supplementary data. The denomination sh-SNHG17 can be introduced elsewhere, such as in the next paragraph or in “cell culture and transfection”*

274 of DoHH2 and Daudi cells (Fig. 3B). Western blotting showed SNHG17 knockdown increased parp

275 protein levels in DoHH2 and Daudi cells (Fig. 3C). In conclusion, SNHG17 knockdown inhibited

276 proliferation and promoted apoptosis of DLBCL cells.

283 Through qPCR and western blot detection, the mRNA and protein levels of caspase-3 decreased and

284 parp mRNA increased in SNHG17-knockdown tumor tissues (Fig. 3H, I)*. Be careful: a decrease in caspase 3 level is not a pro-apoptotic marker. What you really show in picture, considering the molecular weight of 32 kDa, is a decrease in pro-caspase-3 level, which is really a pro-apoptotic marker. Pro-caspase-3 mRNA reduction, on the contrary, has a questionable meaning, but not in an apoptotic sense. Furthermore, PARP as an apoptotic marker must be analyzed at the protein level, where the value considered is the ratio between the caspase-cleaved fragment and the full-length form. To evaluate PARP as an apoptotic marker the wb analysis must be repeated considering both the full length and the cleaved form.*

*Fig 3. DOXO concentration should be indicated at least in the legend*

318 < 0.01), but no significant effect was observed in SNHG17-MUT cells (Fig. 5FI-II). qPCR results

319 showed that compared with PBMC, the expression of miR-34a-5p in DLBCL cells was significantly

320 downregulated (Fig. 5D*~~, G~~*). In addition, SNHG17 knockdown increased the expression of miR-34a-

321 5p in DoHH2 cells *(Fig.5G)*. This result indicated that *miR-34a-321 5p* *~~DoHH2~~* is negatively regulated by SNHG17 *in this cell line*.
